# CSF neurofilament light chain profiling and quantitation in neurological diseases

**DOI:** 10.1093/braincomms/fcae132

**Published:** 2024-04-16

**Authors:** Claire A Leckey, John B Coulton, Tatiana A Giovannucci, Yingxin He, Aram Aslanyan, Rhiannon Laban, Amanda Heslegrave, Ivan Doykov, Francesca Ammoscato, Jeremy Chataway, Floriana De Angelis, Sharmilee Gnanapavan, Lauren M Byrne, Jonathan M Schott, Edward J Wild, Nicolas R Barthelémy, Henrik Zetterberg, Selina Wray, Randall J Bateman, Kevin Mills, Ross W Paterson

**Affiliations:** Dementia Research Centre, UCL Queen Square Institute of Neurology, University College London, London, WC1N 3BG, UK; Translational Mass Spectrometry Research Group, UCL Great Ormond Street Hospital Institute of Child Health, University College London, London, WC1N 1EH, UK; UK Dementia Research Institute at UCL, University College London, London, WC1E 6BT, UK; Department of Neurology, Washington University School of Medicine, Washington University in St Louis, St Louis, MO 63110, USA; Tracy Family SILQ Center, Washington University School of Medicine, Washington University in St Louis, St Louis, MO 63110, USA; Dementia Research Centre, UCL Queen Square Institute of Neurology, University College London, London, WC1N 3BG, UK; UK Dementia Research Institute at UCL, University College London, London, WC1E 6BT, UK; Department of Neurodegenerative Disease, UCL Queen Square Institute of Neurology, University College London, London, WC1N 3BG, UK; Department of Neurology, Washington University School of Medicine, Washington University in St Louis, St Louis, MO 63110, USA; Tracy Family SILQ Center, Washington University School of Medicine, Washington University in St Louis, St Louis, MO 63110, USA; Dementia Research Centre, UCL Queen Square Institute of Neurology, University College London, London, WC1N 3BG, UK; UK Dementia Research Institute at UCL, University College London, London, WC1E 6BT, UK; Department of Neurodegenerative Disease, UCL Queen Square Institute of Neurology, University College London, London, WC1N 3BG, UK; UK Dementia Research Institute at UCL, University College London, London, WC1E 6BT, UK; Department of Neurodegenerative Disease, UCL Queen Square Institute of Neurology, University College London, London, WC1N 3BG, UK; Translational Mass Spectrometry Research Group, UCL Great Ormond Street Hospital Institute of Child Health, University College London, London, WC1N 1EH, UK; Barts and the London School of Medicine and Dentistry, Queen Mary University of London, Blizard Institute, Centre for Neuroscience, London, E1 2AT, UK; Department of Neuroinflammation, Faculty of Brain Sciences, Queen Square Multiple Sclerosis Centre, UCL Queen Square Institute of Neurology, University College London, London, WC1B 5EH, UK; National Institute for Health and Care Research, University College London Hospitals, Biomedical Research Centre, London, W1T 7DN, UK; Department of Neuroinflammation, Faculty of Brain Sciences, Queen Square Multiple Sclerosis Centre, UCL Queen Square Institute of Neurology, University College London, London, WC1B 5EH, UK; National Institute for Health and Care Research, University College London Hospitals, Biomedical Research Centre, London, W1T 7DN, UK; Department of Neurology, Barts Health NHS Trust, London, E1 1BB, UK; Department of Neurodegenerative Disease, UCL Queen Square Institute of Neurology, University College London, London, WC1N 3BG, UK; Dementia Research Centre, UCL Queen Square Institute of Neurology, University College London, London, WC1N 3BG, UK; Department of Neurodegenerative Disease, UCL Queen Square Institute of Neurology, University College London, London, WC1N 3BG, UK; Department of Neurology, Washington University School of Medicine, Washington University in St Louis, St Louis, MO 63110, USA; Tracy Family SILQ Center, Washington University School of Medicine, Washington University in St Louis, St Louis, MO 63110, USA; UK Dementia Research Institute at UCL, University College London, London, WC1E 6BT, UK; Department of Neurodegenerative Disease, UCL Queen Square Institute of Neurology, University College London, London, WC1N 3BG, UK; Department of Psychiatry and Neurochemistry, Institute of Neuroscience and Physiology, The Sahlgrenska Academy at the University of Gothenburg, Mölndal, 43180, Sweden; Clinical Neurochemistry Laboratory, Sahlgrenska University Hospital, Mölndal, 43180, Sweden; Hong Kong Center for Neurodegenerative Diseases, Clear Water Bay, Hong Kong, China; Wisconsin Alzheimer’s Disease Research Center, University of Wisconsin School of Medicine and Public Health, University of Wisconsin-Madison, Madison, WI53792, USA; Department of Neurodegenerative Disease, UCL Queen Square Institute of Neurology, University College London, London, WC1N 3BG, UK; Department of Neurology, Washington University School of Medicine, Washington University in St Louis, St Louis, MO 63110, USA; Tracy Family SILQ Center, Washington University School of Medicine, Washington University in St Louis, St Louis, MO 63110, USA; Translational Mass Spectrometry Research Group, UCL Great Ormond Street Hospital Institute of Child Health, University College London, London, WC1N 1EH, UK; Dementia Research Centre, UCL Queen Square Institute of Neurology, University College London, London, WC1N 3BG, UK; UK Dementia Research Institute at UCL, University College London, London, WC1E 6BT, UK; Department of Neurology, Darent Valley Hospital, Dartford, Kent, DA2 8DA, UK

**Keywords:** neurofilament light chain, mass spectrometry, neurodegenerative diseases

## Abstract

Neurofilament light chain is an established marker of neuroaxonal injury that is elevated in CSF and blood across various neurological diseases. It is increasingly used in clinical practice to aid diagnosis and monitor progression and as an outcome measure to assess safety and efficacy of disease-modifying therapies across the clinical translational neuroscience field. Quantitative methods for neurofilament light chain in human biofluids have relied on immunoassays, which have limited capacity to describe the structure of the protein in CSF and how this might vary in different neurodegenerative diseases. In this study, we characterized and quantified neurofilament light chain species in CSF across neurodegenerative and neuroinflammatory diseases and healthy controls using targeted mass spectrometry. We show that the quantitative immunoprecipitation–tandem mass spectrometry method developed in this study strongly correlates to single-molecule array measurements in CSF across the broad spectrum of neurodegenerative diseases and was replicable across mass spectrometry methods and centres. In summary, we have created an accurate and cost-effective assay for measuring a key biomarker in translational neuroscience research and clinical practice, which can be easily multiplexed and translated into clinical laboratories for the screening and monitoring of neurodegenerative disease or acute brain injury.

## Teaser

Leckey *et al*. report a new mass spectrometry-based method to capture and quantify neurofilament light (NfL) from CSF. The assay targets a specific tryptic peptide of NfL that is highly abundant across neurodegenerative and neuroinflammatory conditions, providing a reliable quantitation tool with utility across the full spectrum of neurological diseases.

## Graphical Abstract

**Graphical Abstract:**
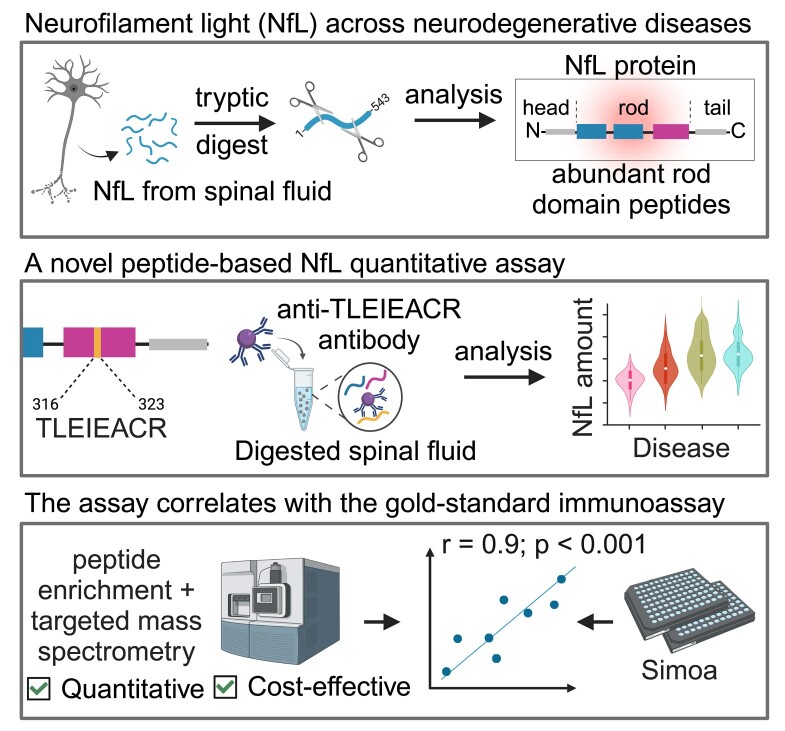
This image was created with BioRender.com.

## Introduction

Neurofilament light chain (NfL) is a structural protein found within large fibre myelinated axons of the human central and peripheral nervous system and is an established fluid biomarker of neuronal injury. It is released into the CSF and blood of healthy individuals, but concentrations are elevated in a range of neurodegenerative and neuroinflammatory diseases. NfL is also significantly increased in non-primary neurological diseases such as hypoxic brain injury^1-3^ and COVID-19.^4,5^ Levels change dynamically in response to acute neuronal injury in traumatic brain injury^6-8^ and multiple sclerosis relapses.^9^ In chronic neurodegenerative diseases, levels correlate with rates of brain atrophy and with clinical progression making it an attractive biomarker of neurodegeneration.^10-13^ It is now widely used for clinical diagnostic purposes (e.g. to help identify neuronal damage) and on a research basis for measuring the clinical response to disease-modifying therapies. It is frequently deployed as an outcome measure in clinical trials across a range of diseases, including Alzheimer’s disease,^14,15^ Huntington’s disease,^16,17^ amyotrophic lateral sclerosis^18-20^ and multiple sclerosis.^21^

NfL concentration in biofluids is currently measured using sandwich immunoassays with antibodies directed against the rod domain of the protein, for example using single-molecule array (Simoa)^22^ or enzyme-linked lectin assay (Ella).^23^ There are several limitations to these immunoassay platforms: scientific, logistic and economic. Firstly, antibody approaches are less reliable for absolute quantitation because they may miss protein oligomerization, post-translational modification, truncation and are liable to epitope masking by auto-antibodies and/or the presence of hetero-aggregates in biofluids.^24,25^ This is because the epitope to which the antibody binds may not be ‘visible’ to the antibody if it is not accessible.^26^ Secondly, the antibody is produced by commercial entities, which limits its characterization and adaptability. Finally, the antibody and assay kits are costly, which may restrict the uptake of NfL into widespread clinical practice especially as alternative tests for NfL are unavailable.

NfL has previously been characterized in CSF,^22^ identifying three main truncated species. One particular peptide that corresponds to the rod Coil 2B domain of the protein is elevated in Alzheimer’s disease and is more likely to be relevant as a biomarker. It is not yet clear whether this peptide has similar relevance across the other neurological diseases. We aimed to profile NfL across a range of neurological diseases and then develop a rapid translational targeted mass spectrometry (MS) assay, using antibodies against tryptic peptides for more reliable quantitation of this particular rod domain of NfL. We aimed to compare this against the Simoa immunoassay and with an alternative independent targeted MS assay. The intended outcome was to generate a cost-effective diagnostic and reference tool that can be used across the full spectrum of neurological diseases.

## Materials and methods

### Participant recruitment and clinical demographics

#### Dementia

We prospectively recruited individuals with suspected neurodegenerative diseases from the specialist cognitive disorders clinics at the National Hospital for Neurology and Neurosurgery (NHNN), Queen Square and Darent Valley Hospital in Kent. Individuals provided consent to donate additional CSF for research prior to undergoing diagnostic lumbar puncture. Consensus criteria were used to classify individuals as probable Alzheimer’s disease,^27^ behavioural variant frontotemporal dementia,^28^ dementia with Lewy bodies,^29^ corticobasal syndrome^30^ and semantic dementia.^31^ All individuals gave informed written consent (ethical permit: 12/LO/1504).

#### Huntington’s disease

Individuals were recruited from the Huntington’s disease multidisciplinary clinic at NHNN to the HD-CSF study.^32^ Expanded CAG repeat in *HTT* was genetically confirmed, and participants consented to research lumbar puncture. Ethical approval was provided by the London Camberwell St Giles Ethics Committee (ref: 186932).

#### Multiple sclerosis

Individuals were recruited from the multiple sclerosis service at the Royal London Hospital to the MS-SMART study. All individuals fulfilled clinical criteria for secondary progressive multiple sclerosis^33^ and gave consent to a research lumbar puncture at University College London (UCL). Ethical approval was provided by the Scotland A Research Ethics Committee (REC: 13/SS/0007).

#### Healthy controls

Controls were spouses or relatives of individuals attending the specialist cognitive disorder clinic at NHNN or who expressed an interest in research through Join Dementia Research (www.joindementiaresearch.nihr.ac.uk). They did not have cognitive concerns and scored >27 on Mini-Mental State Examination, but CSF neurodegenerative biomarker profiles are not available. They were known not to be at risk of a genetic neurodegenerative disease. All individuals provided informed written consent. Ethical approval was given by the Hampstead Ethics Committee (ref: 19/LO/0913).

### Sample collection

All CSF was collected by lumbar puncture according to local clinical standard operating procedures, in polypropylene containers between 09:00 and 15:00 and handled according to standardized predefined standard operating procedures as previously described.^32,34^ All CSF samples were stored at −80°C until analysis.

### Simoa

CSF analysis was performed using the NF-L advantage kit with NfL concentrations measured on a Simoa HDx analyser (Quanterix). Samples were diluted 100×, and the assay performed as per the manufacturer’s protocol. All samples were measured within one experiment and in singlicate with the analyst blinded to clinical status. Internal quality controls (QCs) were monitored to assess the intra-assay and inter-assay coefficients of variation (CV), which were determined to be 5.1–10.5 and 7.8%, respectively.

### IP-MS for profiling of NfL (Washington University in St Louis)

#### Protein-level immunoprecipitation and sample preparation

As previously described,^35^ antibodies targeting Coil 1A/1B (HJ30.13), rod domain Coil 2B (HJ30.4) and tail subdomain (HJ30.11) of NfL were coupled to M270 Epoxy Dynabeads (Invitrogen) and mixed in a 1:1:1 ratio and suspended in 1× phosphate-buffered saline (PBS) to a final concentration of 15 mg/mL coupled beads. CSF samples (350 µL aliquots) were thawed and mixed with 0.5 ng of isotopically labelled internal standard (‘ISTD’, recombinant, ubiquitously labelled ^15^N-NfL, Promise Proteomics) and spiked with 25 µL of a master mix containing detergent (0.5% IGEPAL CA630), chaotropic reagent (5 mM guanidine) and 1× protease inhibitors (Roche cOmplete Protease Inhibitor Cocktail). CSF–ISTD mixes were transferred to a 96-well plate, at which point 30 µL antibody slurry was added to each sample well. Immunoprecipitation (IP) was carried out on a Kingfisher Flex Station (Thermo Scientific), which mixed CSF and antibody slurry for 90 min prior to three sequential washes of the NfL-coupled beads [25 mM triethylammonium bicarbonate (TEABC), 1 mL]. NfL-coupled beads were suspended in 100 µL TEABC (25 mM) for on-bead reduction and alkylation with dithiothreitol (50 mM DTT in 25 mM TEABC, 49 μg spike/sample, 1 h, 1000 rpm, room temperature (RT)] followed by iodoacetamide (IAA, 100 mM in 25 mM TEABC, 50 min, RT, light protected). Trypsin–Lys-C mix (Mass Spec Grade, Promega, in 25 mM TEABC) was then spiked into each sample (400 ng per sample) and incubated for 16 h at 37°C. Resultant tryptic peptides of NfL were isolated and cleaned up via solid-phase extraction (SPE; C-18 TopTip, Glygen). Stationary phase was wetted with 60% acetonitrile (ACN) and 0.1% formic acid (FA; 150 μL) and re-equilibrated with 0.1% FA (three additions of 150 μL). NfL peptides were loaded to TopTip via centrifugation (1109 x *g*, 2 min) and washed by adding 0.1% FA (three additions of 150 μL) to TopTip and centrifugation (1109 x *g*, 2 min). Peptides were eluted by adding 60% ACN and 0.1% FA (two additions of 50 μL) to TopTip and centrifugation (1109 x *g*, 1 min). Cleaned peptide extracts were concentrated by evaporation of eluent *in vacuo* and reconstituted in 25 μL 0.1% FA. Reconstituted samples were centrifuged (21 000 *× g*, 4°C, 15 min), and 21 μL was transferred for analysis via nano-liquid chromatography (LC)–tandem MS (MS/MS).

#### LC–MS/MS

Samples were injected (4.5 µL aliquot) by an M-Class nano-Acquity LC (Waters Corporation) fitted with High Strength Silica (HSS) C18 T3 analytical column (75 µm × 100 µm, 1.8 µm particle diameter). Samples were loaded on column via direct inject at 0.7 µL/min, with mobile phase composition of 99.5% A (0.1% FA) and 0.5% B (ACN, 0.1% FA) from *t* = 0 to 7.5 min. NfL peptides were separated at 0.4 µL/min with the following gradient: *t* = 7.6 min, %A: 99.5, %B: 0.5; *t* = 7.7 min, %A: 94, %B: 6; *t* = 24 min, %A: 66, %B: 34; *t* = 25 min, %A: 5, %B: 95; *t* = 26.99 min, %A: 5, %B: 95; and *t* = 27 min, %A: 99.5, %B: 0.5. NfL peptides were analysed in positive ion mode, spray voltage was 2.2 kV, and ion transfer tube temperature was 275°C. Parallel reaction monitoring was employed for characteristic transitions of endogenous and isotopically labelled (U-15N) NfL peptides (see Supplementary Table 1). All samples were measured with the analyst blinded to clinical status. NfL concentrations were calculated based on ratio to ISTD and reported in picograms per millilitre based on the volume of CSF used for IP.

### Quantitative IP-MS/MS for NfL in CSF (University College London)

#### Tryptic digestion and peptide-level IP of NfL

For each sample, 450 μL of CSF was thawed and spiked with 1 ng of heavy labelled recombinant NfL (Promise Proteomics) prior to protein precipitation by addition of three sample volumes of ice-cold acetone and incubation at −20°C for 16 h. Precipitated protein was pelleted by centrifugation at 14 000 *× g* for 15 min at 4°C. The protein pellet was allowed to air-dry before re-solubilizing in 40 μL digest buffer (8 M urea, 200 mM Tris HCl, pH 8) for at least 30 min. Reduction and alkylation were performed with dithioerythritol (90 μg/sample) at RT with shaking at 1500 rpm for 1 h, followed by incubation with IAA (216 μg/sample) in the dark and at RT with shaking at 1500 rpm for 50 min. Samples were diluted with high-performance LC-grade ultrapure water prior to addition of MS-grade Trypsin–Lys-C to a final concentration of 2 µg/mL, and samples were digested for 16 hours at 37˚C. Post-digestion, and immediately prior to IP, all samples were spiked with 5 μL 6 mM tosyl-L-lysine chloromethyl ketone hydrochloride to inhibit Trypsin–Lys-C activity.

To provide enrichment of NfL, custom rabbit polyclonal antibodies were generated against the NfL_316–323_ peptide (TLEIEACR) from the Coil 2B rod domain (Eurogentec, Belgium). Purified IgG antibodies were coupled to M270 Epoxy Dynabeads™ following the manufacturer’s instructions of the Dynabeads™ Antibody coupling kit (Invitrogen). Coupled beads were resuspended in 1× PBS at 10^9^ beads/mL. Fifty microlitres of antibody-coupled beads were added to each sample digest and incubated with rotation for 1.5 h at RT (20°C). The NfL-coupled beads were washed three times in 0.5 mM CHAPS (3-[(3-cholamidopropyl)dimethylammonio]-1-propane**s**ulfonate) in PBS before eluting in 50 μL 1% FA (in 0.5 mM CHAPS) with shaking at 1000 rpm for 6 min. Eluates containing the enriched NfL peptide were transferred to a 96-well sample collection plate fitted at the bottom with a magnet (SISCAPA Assay Technologies) ready for ultra-performance LC (UPLC)-MS/MS analysis.

#### UPLC–MS/MS

Analysis of NfL was performed on an Acquity™ I-Class PLUS UPLC coupled to a Xevo™ TQ-XS triple quadrupole mass spectrometer operated in positive electrospray ionization (ESI^+^) mode (Waters Corporation). Samples (15 μL) were injected onto an Acquity Premier peptide ethylene bridged hybrid (BEH) C18 analytical column (300 Å, 1.7 μm, 2.1 × 50 mm) held at 50°C. Initial mobile phase composition was set to 97% A (0.1% FA) and 3% B (ACN, 0.1% FA) at 0.2 mL/min. Chromatographic separation was performed over the following 16-min gradient: initial conditions were held until 0.2 min after which B was linearly increased to 35% by 11 min. To wash the column, B was increased to 100% over a 1-min linear gradient and held for 1.8 min at an increased flow rate of 0.6 mL/min before returning the system to initial conditions and re-equilibrating the column for 2.2 min. Mass spectrometer settings were as follows: 300°C desolvation temperature, 600 L/h desolvation gas flow, 2.5 kV capillary voltage, 150 L/h cone gas and 0.15 mL/min collision gas.

Samples were injected in a randomized order, with high and low QC samples analysed every 20 injections to monitor system performance. All samples were measured with the analyst blinded to clinical status.

All data were acquired by scheduled multiple reaction monitoring (MRM), with one quantifier and one qualifier ion monitored for each transition (see Supplementary Table 1). Raw acquisition data were imported and processed in Skyline (version 22.2, MacCoss Lab, University of Washington). Integrated peak areas and peak area ratios were exported into Microsoft Excel. NfL concentrations in picograms per millilitre were calculated based on an eight-point calibration curve as previously described.^35^

### Statistical analysis

Statistical analysis was performed in IBM Statistical Package for the Social Sciences (SPSS) Statistics (version 26) and R (version 4.2.2). Normality of the data was assessed visually by Q–Q plots and numerically by the Shapiro–Wilk or Kolmogorov–Smirnov tests where appropriate. Due to cohort (*n* = 85) and disease group (*n* = 6–19) size, the Spearman ranked correlation was used to assess the relationship between analytical methods for the overall cohort and within clinical diagnostic groups. To identify significant differences in NfL_316–323_ (TLEIEACR) concentrations between clinical groups, non-parametric Mann–Whitney U-tests were performed.

To assess agreement between quantitative methods, IP-MS/MS (UCL) and Simoa, Bland–Altman testing was performed.^36,37^ The calculated differences between methods were assessed for normality, and data found to reject the null hypothesis were log transformed prior to the Bland–Altman analysis.^38^

To evaluate and compare method performance, confusion matrix analysis was performed based on current age-related clinical cut-off values for normal CSF NfL concentrations. Actual class positive or negative assignment was based on clinical diagnosis of study participants at time of sampling as those with a neurodegenerative disease or as a healthy control, respectively. Predictive class positive or negative assignment was based on current age-related clinical cut-off concentrations for normal CSF NfL as specified in Yilmaz *et al*.^39^

## Results

### Profiles of NfL in CSF across neurodegenerative and neuroinflammatory disease groups

**Figure 1:**
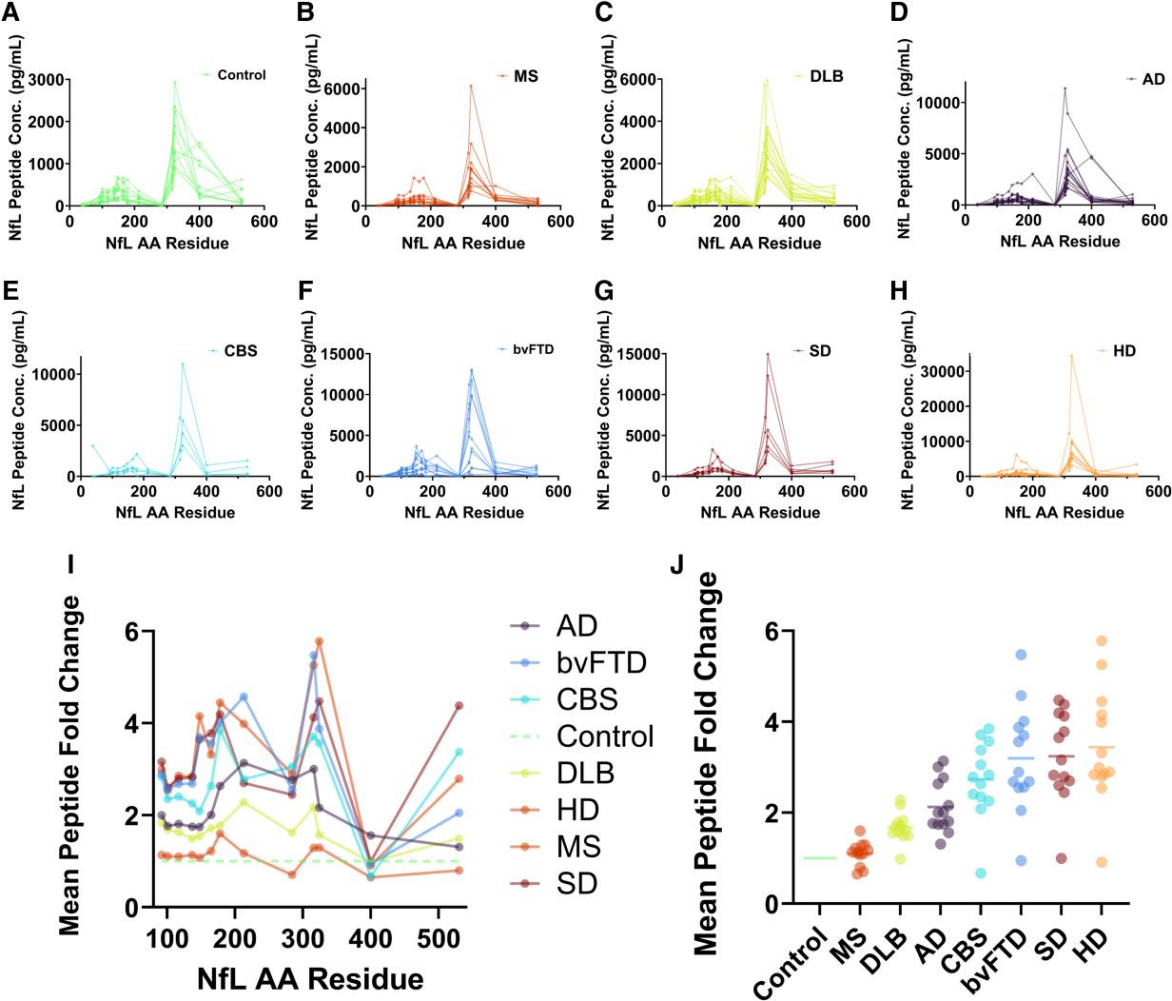
**NfL profiles in CSF across neurodegenerative disease groups show greatest detection of NfL species from the Coil 2B domain.** NfL concentrations determined by IP–MS (WashU) are plotted by the peptide amino acid (AA) residue and represent NfL species located in Coil 1A (NfL_93–124_), Coil 1B (NfL_138–234_) and Coil 2B (NfL_281–396_) of the mid-rod domain and subdomain B (NfL_444–543_) of the C-terminal tail domain. (**A–H**) Traces represent individual NfL peptide concentrations, with control or disease states detailed by panel legends. (**I**) Mean fold change profiles across NfL peptides for each disease state compared to control and (**J**) mean NfL peptide fold change (compared to controls) for each disease state (horizontal bars represent mean fold change and dots represent fold change for each monitored peptide). Detected peptide sequences by NfL domain and amino acid residues are specified in Supplementary Table 1. Ad, Alzheimer’s disease; bvFTD, behavioural variant frontotemporal dementia; CBS, corticobasal syndrome; DLB, dementia with Lewy bodies; HD, Huntington’s disease; IP–MS, immunoprecipitation–mass spectrometry; MS, multiple sclerosis; NfL, neurofilament light chain; SD, semantic dementia; WashU, Washington University in St Louis.

To map the relative amounts of protein fragments (proteoforms) of NfL in CSF, protein-level IP–MS analysis was performed at Washington University in St Louis (WashU) with the analyst blinded to clinical status. For all clinical groups, a total of 13 NfL peptides were detected across the following structural domains: Coil 1A (*n* = 3), Coil 1B (*n* = 5), Coil 2B (*n* = 3), C-terminal tail subdomain A (*n* = 1) and tail subdomain B (*n* = 1). The resulting CSF NfL profiles for healthy controls and the studied neurodegenerative and neuroinflammatory diseases are shown in Fig. 1, with the greatest detection for the neighbouring Coil 2B peptides NfL_324–331_ (GMNEALEK) and NfL_316–323_ (TLEIEACR) observed in all clinical groups. Some notable but subtle differences were observed at the N- and C-terminus peptides (Fig. 1). NfL_324–331_ and NfL_530–540_ relative profiles by disease are shown in Supplementary Fig. 1 to determine potential qualitative differences in NfL proteolysis by pathology.

### Comparison of NfL targeted MS methods to Simoa

The study cohort demographics and NfL measures across analytical assays by clinical group are summarized below in Table 1, with NfL peptide-level IP–MS/MS (UCL) and NfL protein-level IP–MS (WashU) determined by detection of the NfL_316–323_ peptide (TLEIEACR).

**Table 1:** Clinical cohort demographics and neurofilament light measures across assays

	Age at LP (years)	% male	NfL Simoa (pg/mL)	NfL IP–MS/MS^a^ (pg/mL)	NfL IP–MS^b^ (pg/mL)
Controls (*n* = 10)	64 (62–76)	70	617.2 (417.9–735.6)	778.4 (607.3–1208.5)	722.6 (526.5–1265.0)
Alzheimer’s disease (*n* = 15)	66 (60–69)	53	1007.0 (700.0–1317.1)	1743.7 (1342.2–2126.9)	1807.8 (1321.3–1990.2)
bvFTD (*n* = 11)	62.5 (60–67)	82	2793.9 (476.9–3714.2)	2665.4 (892.8–4655.1)	4928.0 (581.2–8215.3)
CBS (*n* = 4)	61.5 (57–66)	75	1197.1 (1071.5–2698.5)	1699.2 (1656.3–4137.3)	2490.3 (1841.7–4940.9)
Dementia with Lewy bodies (*n* = 19)	67 (61–70)	74	882.9 (703.3–1099.9)	1576.2 (1234.3–2009.2)	1605.1 (1039.6–1870.5)
Huntington’s disease (*n* = 10)	57 (44–60)	70	2510.8 (2108.8–3218.4)	4090.7 (2883.0–4770.9)	3674.6 (2958.5–4793.9)
Multiple sclerosis (*n* = 10)	56 (49–61)	40	628.4 (523.5–932.2)	976.9 (698.5–1480.0)	906.6 (584.3–1268.1)
Semantic dementia (*n* = 6)	62 (56–68)	83	1322.9 (1046.9–2039.4)	2670.1 (2290.0–3139.4)	2475.7 (1601.6–5826.9)

Data are represented as median (interquartile range). bvFTD, behavioural variant frontotemporal dementia; CBS, corticobasal syndrome; LP, lumbar puncture; NfL, neurofilament light chain; Simoa, single-molecule array.

^a^IP–MS/MS method developed and run at UCL (University College London).

^b^IP–MS method developed and run at WashU (Washington University in St Louis).

Relative differences in mean NfL concentration across groups remained the same for all assays, with lowest–highest mean NfL concentrations observed in healthy controls, multiple sclerosis, dementia with Lewy bodies, Alzheimer’s disease, semantic dementia, corticobasal syndrome, behavioural variant frontotemporal dementia and Huntington’s disease (Supplementary Fig. 2).

**Figure 2:**
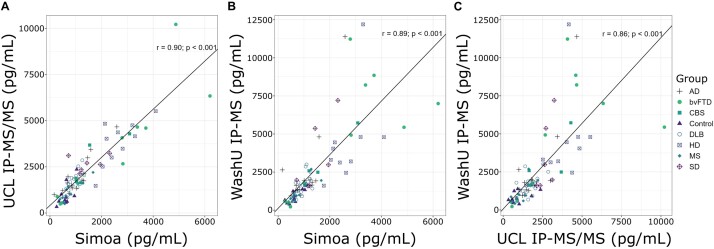
**NfL concentration measured by Simoa strongly correlates with the IP–MS methods for NfL_316–323_ peptide (TLEIEACR) developed at each study centre**. Spearman’s correlations between NfL concentrations as measured by (**A**) IP–MS/MS (UCL) and Simoa (*r* = 0.90, *P* < 0.001), (**B**) IP–MS (WashU) and Simoa (*r* = 0.89, *P* < 0.001) and (**C**) IP–MS/MS (UCL) and IP-MS (WashU; *r* = 0.86, *P* < 0.001) are shown. NfL data plotted for both MS assays represent NfL_316–323_ (TLEIEACR). Strong correlation is observed across all method comparisons, with the highest correlation observed between IP–MS/MS (UCL) and Simoa methods. Ad, Alzheimer’s disease; bvFTD, behavioural variant frontotemporal dementia; CBS, corticobasal syndrome; DLB, dementia with Lewy bodies; HD, Huntington’s disease; IP–MS/MS, immunoprecipitation–tandem mass spectrometry; IP–MS, immunoprecipitation–mass spectrometry; MS, multiple sclerosis; NfL, neurofilament light chain; SD, semantic dementia; Simoa, single-molecule array; UCL, University College London; WashU, Washington University in St Louis.

From all the NfL peptides measured during CSF profiling, NfL_316–323_ peptide concentrations quantified by IP–MS/MS (UCL) and IP–MS (WashU) methods were found to correlate most strongly with Simoa measures, *r* = 0.90 (*P* < 0.001) and *r* = 0.89 (*P* < 0.001), respectively (Fig. 2A and B). NfL_316–323_ concentrations measured by the different targeted MS approaches across the study centres were also found to strongly correlate, *r* = 0.86 (*P* < 0.001) (Fig. 2C). Correlations between IP–MS (WashU) and Simoa for all detected NfL peptides are provided in Table 2.

**Table 2:** Correlation of IP–MS measured NfL concentration to Simoa by peptide sequence and protein domain

Structural domain	Peptide sequence	Amino acid residues	Correlation to Simoa (Spearman coefficient)
Coil 1A	AQLQDLNDR	92–100	0.78
	FASFIER	101–107	0.77
	VLEAELLVLR	117–126	0.80
Coil 1B	ALYEQEIR	137–144	0.77
	LAAEDATNEK	148–157	0.76
	EGLEETLR	165–172	0.77
	YEEEVLSR	178–185	0.68
	IDSLMDEISFLK	213–224	0.76
Coil 2B	FTVLTESAAK	284–293	0.63
	TLEIEACR	316–323	0.89
	GMNEALEK	324–331	0.86
Tail subdomain A	LSFTSVGSITSGYSQSSQVFGR	400–421	0.11
Tail subdomain B	VEGAGEEQAAK	530–540	0.51

IP–MS, immunoprecipitation–mass spectrometry; NfL, neurofilament light chain; Simoa, single-molecule array.

**Figure 3:**
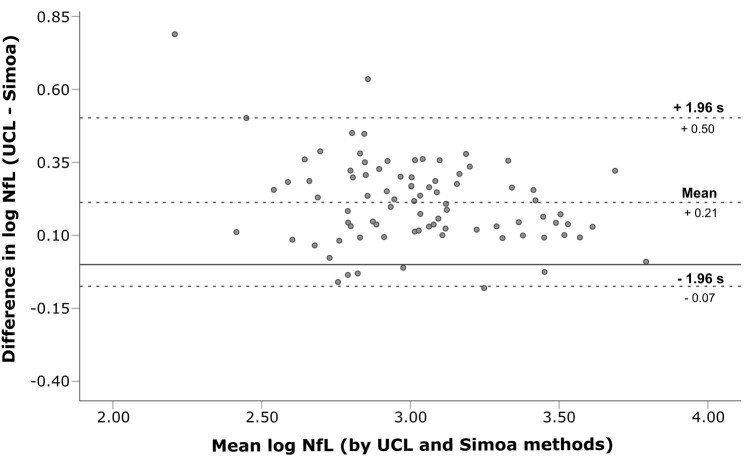
**Evaluation of method agreement between IP–MS/MS (UCL) and Simoa NfL measures demonstrates good agreement between assays.** Bland–Altman analysis to show differences in log-transformed NfL concentrations measured by IP–MS/MS (UCL) and Simoa against the mean log NfL concentration of the methods, with the line of equality represented by the solid line. Bias between the methods is represented as the mean of the difference (+0.21), with the upper and lower 95% LoA plotted as +1.96 s (+0.50) and −1.96 s (−0.07), respectively. IP–MS/MS, immunoprecipitation–tandem mass spectrometry; NfL, neurofilament light chain; s, standard deviation; Simoa, single-molecule array; UCL, University College London.

To better determine the agreement between Simoa and the IP–MS/MS (UCL) method, Bland–Altman analysis was performed on log-transformed NfL concentrations (Supplementary Fig. 3E), with limits of agreement (LoA) defined as the mean difference ± 1.96 × the standard deviation of the differences (s; Fig. 3). Visual assessment of the Bland–Altman plot suggests good agreement between IP–MS/MS (UCL) and Simoa methods. NfL concentrations measured by IP–MS/MS (UCL) were on average 59% higher than those measured by Simoa. Further comparison of all methods showed good agreement between the IP–MS/MS (UCL) and IP–MS (WashU) methods (Supplementary Fig. 3C and D) and the IP–MS (WashU) method with Simoa (Supplementary Fig. 3A and B).

### Clinical group differences determined by Simoa are replicated by quantitative NfL_316–323_ IP–MS/MS (UCL)

**Figure 4:**
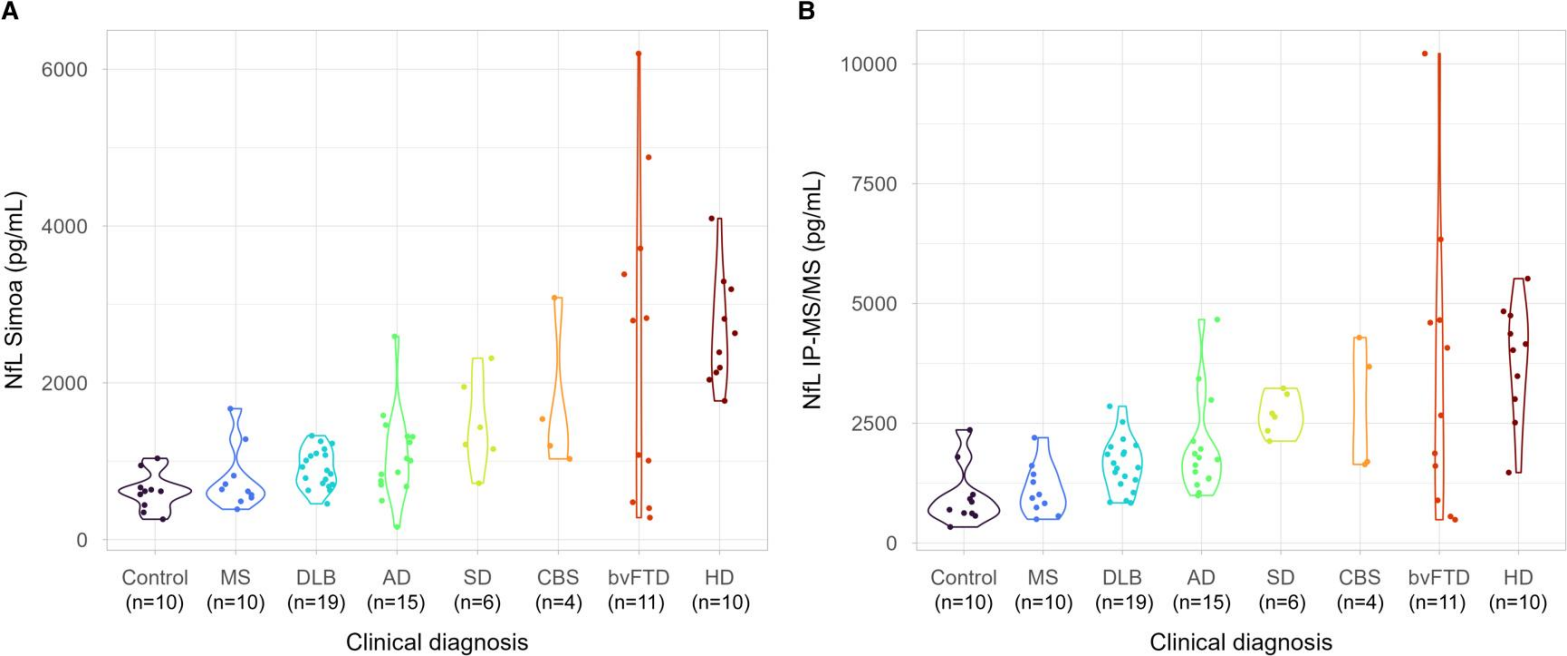
**Relative differences in NfL concentration between clinical groups are conserved when measured by Simoa or IP–MS/MS (UCL).** The relative differences in CSF NfL concentration between clinical groups as measured by (**A**) Simoa were found to be conserved when compared to (**B**) measurement of the Coil 2B NfL_316–323_ (TLEIEACR) peptide by IP–MS/MS (UCL). Significant differences in NfL concentrations between clinical diagnostic groups were determined by Mann–Whitney U-tests. For both NfL assays, all neurodegenerative diseases except the multiple sclerosis group were found to be significantly different to healthy controls: dementia with Lewy bodies (*P* < 0.01), Alzheimer’s disease (*P* ≤ 0.01), semantic dementia (*P* = 0.001), corticobasal syndrome (*P* < 0.05), behavioural variant frontotemporal dementia (*P* < 0.05) and Huntington’s disease (*P* < 0.0005). Ad, Alzheimer’s disease; bvFTD, behavioural variant frontotemporal dementia; CBS, corticobasal syndrome; DLB, dementia with Lewy bodies; HD, Huntington’s disease; IQT, interquartile range; MS, multiple sclerosis; NfL, neurofilament light chain; SD, semantic dementia.

To further assess agreement between quantitative methods, differences in NfL concentrations across clinical diagnosis groups were evaluated for IP–MS/MS (UCL) and Simoa measures (Fig. 4). The same significant differences in NfL concentrations measured by Simoa (Fig. 4A) and IP–MS/MS (Fig. 4B) were observed between healthy controls and all neurodegenerative diseases, except for the multiple sclerosis group. The most significant difference in NfL concentration between healthy controls and disease was observed for the Huntington’s disease group (*P* < 0.0005; *n* = 10) for both IP–MS/MS and Simoa methods.

Spearman ranked correlation was used to assess the relationship between analytical methods within clinical diagnostic groups. We found that the correlation strength of Simoa and IP–MS/MS (UCL) methods varied by disease group (Supplementary Fig. 4). Very strong correlations were seen in behavioural variant frontotemporal dementia (*r* = 0.94, *P* < 2.2e^−16^), multiple sclerosis (*r* = 0.85, *P* = 0.004), dementia with Lewy bodies (*r* = 0.81, *P* = 3e^−5^), corticobasal syndrome (*r* = 0.8, *P* = 0.3) and Alzheimer’s disease (*r* = 0.79, *P* = 7e^−04^). Strong correlations were seen in Huntington’s disease (*r* = 0.64, *P* = 0.05) and controls (*P* = 0.56, *P* = 0.1). The weakest correlation was seen in semantic dementia (*r* = 0.37, *P* = 0.1; slope = 0.23).

Finally, we evaluated the clinical sensitivity, specificity and positive and negative predictive values of the IP–MS/MS (UCL) assay compared to Simoa. This analysis determined the IP–MS/MS (UCL) assay to provide equivalent positive and negative predictive values as Simoa, but greater sensitivity, accuracy and precision (Supplementary Fig. 5).

## Discussion

We have characterized NfL in CSF across a range of neurodegenerative and neuroinflammatory diseases and show that the most abundant NfL peptides identifiable by MS were from Coil 2B of the protein (amino acids 281–396). Having confirmed this, we have developed a targeted MRM method (IP–MS/MS) to quantitate NfL in CSF. Our results are highly correlated with the current ‘gold standard’ clinically accredited Simoa immunoassay that is used in clinical research to quantitate NfL in CSF and plasma to support diagnosis and monitor disease progression.^40^

As disease-modifying therapies are now widely used in multiple sclerosis, have recently been licenced in Alzheimer's disease, with more FDA approvals likely^41^ and are in trial in other neurodegenerative diseases, it is critically important that we have access to biomarkers that support diagnosis and can be used to monitor target engagement. We show that this novel multiplexable assay can reliably and inexpensively quantitate NfL across a broad spectrum of neurodegenerative and neuroinflammatory diseases and has similar clinical diagnostic utility.

We used a large cohort of well-characterized participants from a single specialist centre. These findings were replicated between two independent research institutions, using autonomously developed methods, in academic labs in the UK and USA. One used peptide-level IP and the other used protein-level IP, both delivering similar results. We show that the targeted IP–MS/MS method has potential for translation into a clinical assay on a MS platform that is widely available in routine clinical laboratories. Although the upfront cost of a MS is high, the test itself costs around 12 USD, making it up to 10-fold cheaper than Simoa to run and could be multiplexed with other MS assays. It could also be used to value assign reference materials in further standardization projects.

Based on previous work,^26,35^ we suspected that existing immunoassays bind and quantitate the rod domain of the NfL protein. We set out to establish whether the relevant abundance of this NfL species was consistent across diseases. Using in-house antibodies that bind to different regions of the NfL protein, we were able to obtain comprehensive antibody pull-down in human CSF. We show that the most abundant NfL peptides identifiable by MS were from peptides corresponding to Coil 2B of the protein (amino acids 281–396). Importantly, this region is consistently increased across neurodegenerative and neuroinflammatory diseases and controls, showing that we are likely to be measuring the same NfL species across very different diseases, at unselected stages of disease.

Although the relative concentrations of NfL were consistent between the two assays (with some exceptions, discussed below), we noted with interest a ∼2-fold increase in mean CSF NfL_316–323_ concentration measured by MS compared to Simoa (Supplementary Fig. 2), which may reflect the detection of NfL forms that are missed by Simoa. We already know that different truncations are found in CSF,^35^ and that these truncations are dimerized, making them difficult to detect by sandwich immunoassays.^42^ More specifically, it may be that most NfL species in CSF are dimers with truncated N- and C-termini^42^; such a dimer would be quantified as one monomer using a sandwich immunoassay and two monomers using MS-based assays, potentially explaining our results.

There are few studies that directly compare protein and peptide antibody approaches for the same protein, but it has been suggested that peptide antibodies are more sensitive^43^ and specific,^44^ but with antibody efficiency being dependent on the structure of the protein or peptide in question.^45^ It is noteworthy that both approaches for purifying NfL, using either peptide or protein-level IP, delivered comparable results. This implies that NfL can, in principle, be effectively captured by either approach.

We observed some subtle disease-specific differences in correlations. The weakest correlation and notably different correlation slope between assays were observed in semantic dementia, a language-led variant of frontotemporal dementia with a strong clinicopathological correlation with TDP-43 type C pathology.^46^ This raises the possibility that the CSF pool of NfL in semantic dementia could represent different NfL truncations or different sub-populations of truncations.

We also observed some differences in peptide fold change stratification by disease state (Fig. 1I and J). Generally, fold change ranking and stratification were consistent by disease state from Coil 1 to Coil 2 peptides (including immunoassay analogues NfL_316–323_ and NfL_324–331_). Mean fold change ranking by disease state was identical to a larger ELISA-based meta-analysis.^10^ Stratification and disease state fold change rank changed for C-terminal tail subdomain B peptide (NfL_530–540_), where semantic dementia, corticobasal syndrome, Huntington’s disease, and behavioural variant frontotemporal dementia show highest fold change (ranked third, fourth, first and second, respectively, by NfL_316–323_ fold change stratification). This could imply variability in proteolysis of the tail subdomain relative to processing of Coil 2 of NfL and warrants further investigation.

A major strength of this study is that we developed a targeted method for NfL in one academic laboratory and then validated it using an independent method on a different mass spectrometer in a different institution. We used the same cohort of participants from CSF aliquots collected and handled identically. We were able to show a high level of correlation of results between centres, methods and equipment. This demonstrates that IP targeted MS is an extremely sensitive, specific and reproducible method for quantitating NfL, with potential for simple translation into validated clinical laboratories—for example, triple quadrupole platforms are found in many clinical laboratories in the UK, making it potentially translatable into clinical practice.

This study is not free from limitations. Our clinical cohorts are not demographically balanced; however, this reflects the rarity of selected conditions and the different ages at which these diseases commonly present. Methodologically, the use of different methods to report NfL concentrations between the IP–MS and IP–MS/MS assays limits the extent to which the two can be quantitatively compared.

In summary, we describe a novel assay to quantitate NfL, a widely used fluid biomarker that tracks disease activity and neurodegeneration.^47^ Our assay requires an antibody for tryptic peptide pull-down, but is less dependent on epitope visibility and other potential analytical challenges of current immunoassays such as the documented presence of auto-antibodies against neurofilaments due to their release into CSF during neurodegeneration^48^ and the masking of the epitopes of Uman antibodies used in immunoassays for NfL.^26^ Specifically, peptide-based MS assays overcome such issues due to the sample preparation pipeline prior to analysis, which utilizes trypsin to digest proteins. This makes it inherently more suitable for absolute protein quantitation as the peptides detected result from tryptic cleavage of all forms of NfL. The IP–MS/MS (UCL) method can be run rapidly in 16 min on a triple quadrupole mass spectrometer, such as those currently used in clinical practice and at a lower cost of ∼£10 (∼12 USD) per sample compared to ∼£80/sample by immunoassay. This assay could also be multiplexed with other targeted MRMs to allow quantitation of several proteins in tandem at low cost and on a high-throughput basis.

## Supplementary Material

fcae132_Supplementary_Data

## Data Availability

The data sets generated during the current study are available from the corresponding author upon reasonable request.
