# Supplementary material for: CSF neurofilament light chain profiling and quantitation in neurological diseases
Source: Brain Commun. 2024 Apr 16;6(3):fcae132. doi: 10.1093/braincomms/fcae132 (PMC11069115; doi:10.1093/braincomms/fcae132)
Supplement: fcae132_Supplementary_Data [file fcae132_supplementary_data.pdf]

Supplementary material for *"Cerebrospinal fluid neurofilament light chain profiling and quantitation in neurological diseases"*

Supplementary Table 1. Monitored peptides and ion transitions for endogenous NfL and the following heavy isotope labelled internal standards: ubiquitously labelled ^{15}N- NfL (WashU) or $^{13}C_6$, ^{15}N-labelled Arg/Lys NfL (UCL).

NfL domain	Peptide sequence	Amino acid residues	Precursor ion (m/z)	Precursor charge state (z)	Product ion (type)	Product ion (m/z)
Coil 1A	AQLQDLNDR	92-100	536.7727	2	y7+<br>y5+	873.4425<br>632.2998
	AQLQDLNDR [^{15}N ISTD]		544.2505	2	y7+<br>y5+	885.4069<br>641.2731
	FASFIER	101-107	435.2294	2	y5+<br>y6+<br>y4+	651.3461<br>722.3832<br>564.3140
	FASFIER [^{15}N ISTD]		440.2146	2	y5+<br>y6+<br>y4+	659.3223<br>731.3565<br>571.2933
	VLEAELLVLR	117-126	577.8606	2	y8+<br>y7+<br>y6+	942.5619<br>813.5193<br>742.4822
	VLEAELLVLR [^{15}N ISTD]		584.3415	2	y8+<br>y7+<br>y6+	953.5292<br>823.4896<br>751.4555
Coil 1B	ALYEQEIR	137-144	511.2693	2	y6+<br>y4+	837.4101<br>545.3042
	ALYEQEIR [^{15}N ISTD]		517.2515	2	y6+<br>y4+	847.3805<br>553.2805
	LAAEDATNEK	148-157	531.2591	2	y8+<br>y9+	877.3898<br>948.4269
	LAAEDATNEK [^{15}N ISTD]		537.2413	2	y8+<br>y9+	887.3601<br>959.3943
	EGLEETLR	165-172	473.7456	2	y4+<br>y3+	518.2933<br>389.2507
	EGLEETLR [^{15}N ISTD]		479.2293	2	y4+<br>y3+	525.2725<br>395.2329
	YEEEVLSR	178-185	512.7509	2	y6+<br>y3+	732.3886<br>375.2350
	YEEEVLSR [^{15}N ISTD]		518.2346	2	y6+<br>y3+	741.3620<br>381.2173
	IDSLMDEISFLK	213-224	705.8629	2	y8+<br>y10+	982.4914<br>1182.6075
	IDSLMDEISFLK [^{15}N ISTD]		712.3436	2	y8+<br>y10+	991.4647<br>1193.5749
Coil 2B	FTVLTESAAK	284-293	533.7926	2	y8+<br>y7+	818.4618<br>719.3934
	FTVLTESAAK [^{15}N ISTD]		539.2763	2	y8+<br>y7+	827.4351<br>727.3697
	TLEIEACR	316-323	496.2475	2	y6+<br>y5+<br>y4+	777.3560<br>648.3134<br>535.2293
	TLEIEACR [^{15}N ISTD]		501.7312	2	y6+<br>y5+<br>y4+	786.3293<br>656.2897<br>542.2086
	TLEIEACR [$^{13}C_6$, ^{15}N ISTD]		501.2516	2	y6+<br>y5+	787.3642<br>658.3216

	GMNEALEK	324-331	446.2157	2	y6+<br>y4+<br>y5+	703.3621<br>460.2766<br>589.3192
	GMNEALEK [^{15}N ISTD]		451.2008	2	y6+<br>y4+<br>y5+	711.3384<br>465.2617<br>595.3014
Tail Subdomain A	LSFTSVGSITSGYSQSSQVFGR	400-421	765.7132	3	y9+<br>y8+<br>y7+	995.4905<br>908.4585<br>780.3999
	LSFTSVGSITSGYSQSSQVFGR [^{15}N ISTD]		774.6866	3	y9+<br>y8+<br>y7+	1009.4490<br>921.4199<br>791.3673
Tail Subdomain B	VEGAGEEQAAK	530-540	544.7646	2	y9+<br>y7+	860.4108<br>732.3523
	VEGAGEEQAAK [^{15}N ISTD]		551.2453	2	y9+<br>y7+	871.3782<br>741.3256

Abbreviations: ISTD, internal standard; NfL, neurofilament light chain; UCL, University College London; WashU, Washington University in St Louis.

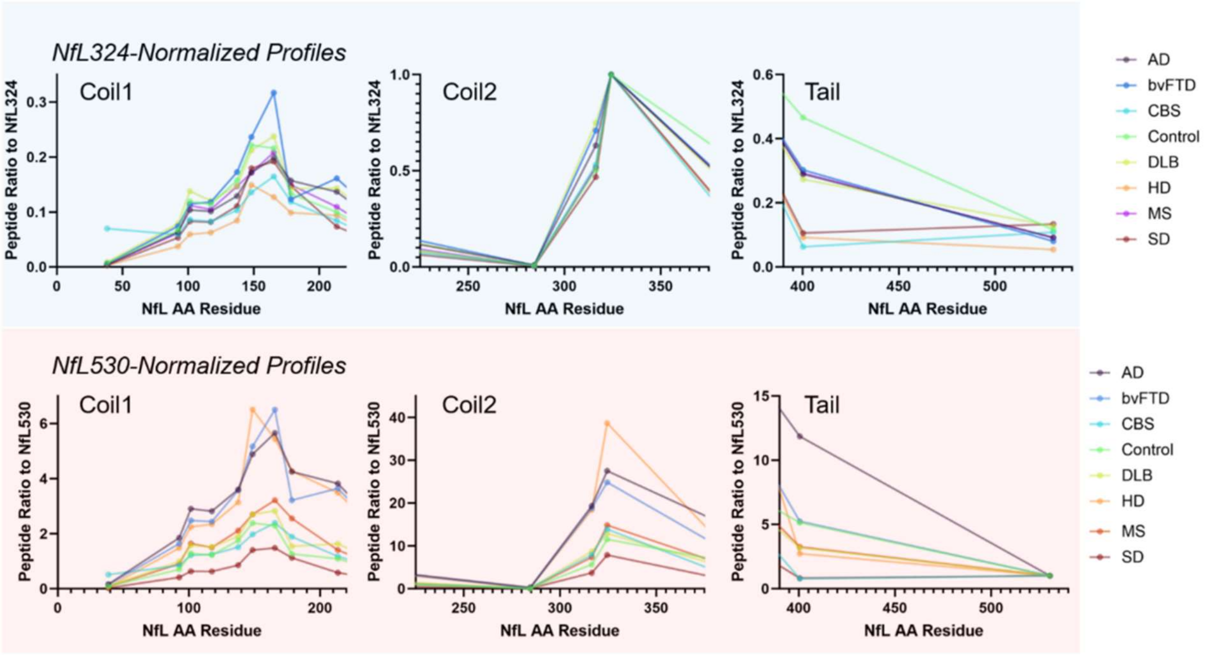

		Rank							
		Control	AD	bvFTD	CBS	DLB	SD	MS	HD
Coil1	92.324	3	5	2	6	1	7	4	8
	101.324	2	5	3	6	1	7	4	8
	117.324	3	5	2	6	1	7	4	8
	137.324	2	5	1	7	4	6	3	8
	148.324	2	6	1	8	3	4	5	7
	165.324	3	6	1	7	2	5	4	8
	178.324	6	2	5	7	4	3	1	8
Coil2	213.324	5	2	1	7	3	8	4	6
	284.324	5	1	4	3	2	6	7	8
	316.324	7	3	2	4	1	8	5	6
	324.324	--	--	--	--	--	--	--	--
	400.324	1	2	3	8	5	6	4	7
Tail	530.324	3	7	6	4	1	2	5	8

Coil1	92.530	7	1	2	6	4	8	5	3
	101.530	6	1	2	7	5	8	4	3
	117.530	7	1	3	6	5	8	4	2
	137.530	6	2	1	7	5	8	4	3
	148.530	6	3	2	7	5	8	4	1
	165.530	7	3	1	6	5	8	4	2
	178.530	7	1	3	5	6	8	4	2
Coil2	213.530	7	3	1	6	5	8	4	2
	284.530	6	1	2	4	5	8	7	3
	316.530	7	3	1	5	4	8	6	2
	324.530	7	2	3	5	6	8	4	1
	400.530	2	1	3	8	5	7	4	6
Tail	530.530	--	--	--	--	--	--	--	--

Supplementary Figure 1. $NfL_{324-331}$ and $NfL_{530-540}$-relative profiles, ratios, and ranks by peptide for each clinical disease state.

NfL peptide concentrations were divided by $NfL_{324-331}$ and $NfL_{530-540}$ concentrations to generate profiles normalized to prominent Coil 2B and Tail subdomain peptides. NfL324 and NfL530-relative profiles are separated into 3 segments corresponding to Coil 1, Coil 2, and Tail subdomain. Resultant NfL324 and NfL530-relative profiles were used to compare peptide rank by disease state (tables at bottom). Profiles relative to NfL Coil 2 and Tail domains were generated to determine potential qualitative differences in NfL proteolysis by pathology. Abbreviations: NfL, neurofilament light chain; AA, amino acid; AD, Alzheimer's disease; bvFTD, behavioural variant frontotemporal dementia; CBS, corticobasal syndrome; DLB, dementia with Lewy bodies; HD, Huntington's disease; MS, multiple sclerosis; NfL, neurofilament light chain; SD, semantic dementia.

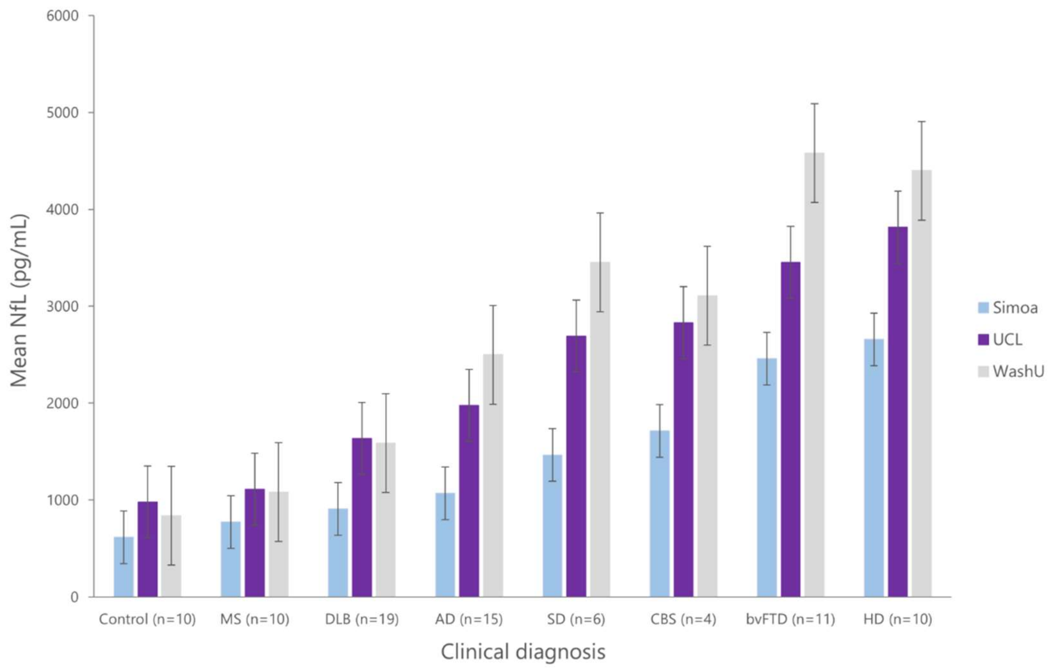

Supplementary Figure 2. Mean NfL concentrations across clinical groups as measured by Simoa and $NfL_{316-323}$ peptide by mass spectrometry methods.

Mean NfL concentrations are plotted for each clinical diagnostic group and NfL assay. Error bars represent standard error of the mean (SEM). Abbreviations: NfL, neurofilament light chain; MS, multiple sclerosis; DLB, dementia with Lewy bodies; AD, Alzheimer's disease; SD, semantic dementia; CBS, corticobasal syndrome; bvFTD, behavioural variant frontotemporal dementia; HD, Huntington’s disease; Simoa, single-molecule array; UCL, University College London; WashU, Washington University in St Louis.

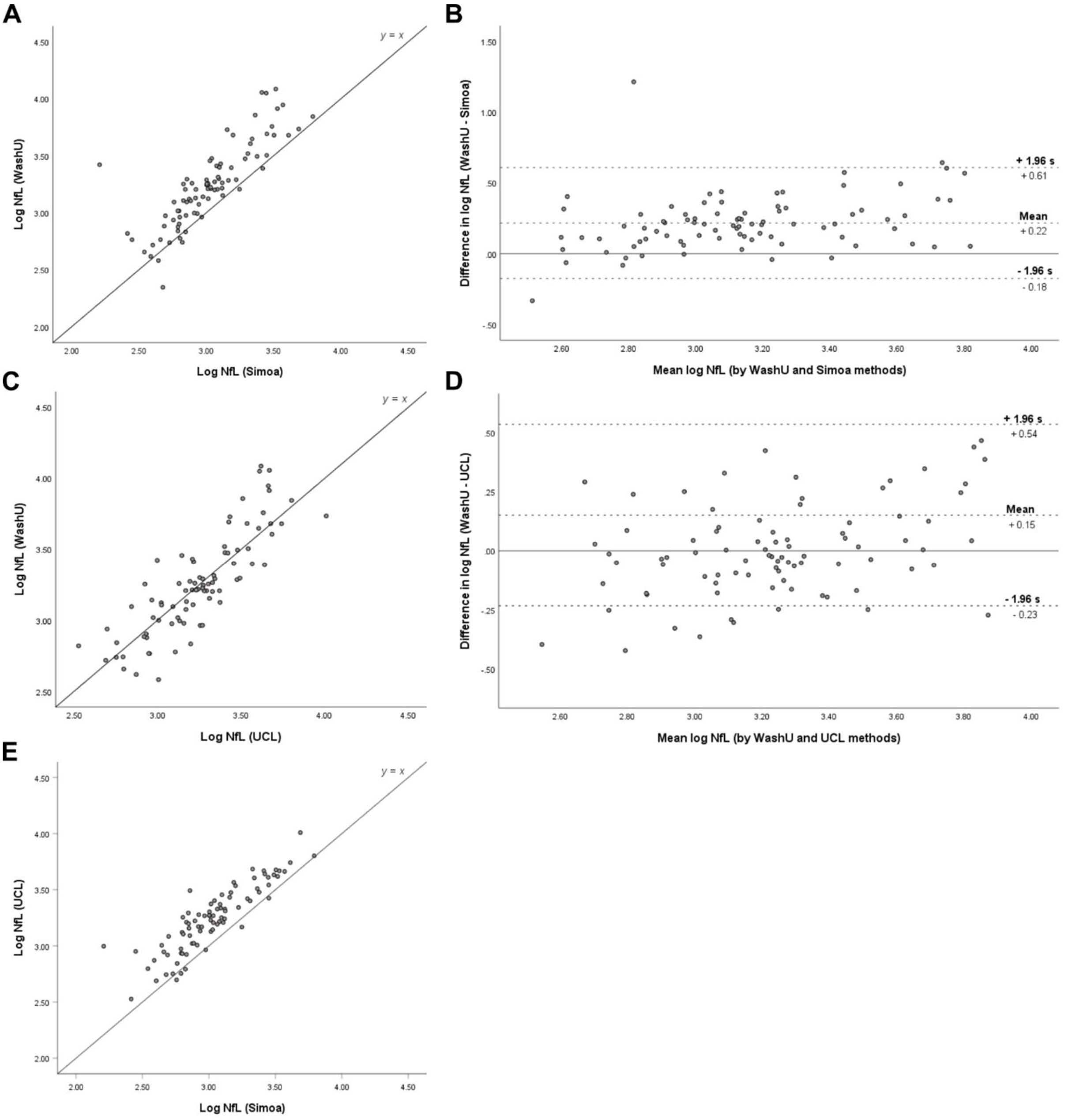

Supplementary Figure 3. Evaluation of method agreement of IP-MS (WashU) with Simoa and IP-MS/MS (UCL) NfL measures demonstrate good agreement between assays.

NfL concentration after log transformation and subsequent Bland-Altman analysis plots are shown for comparison of the following methods: (**A**-**B**) $NfL_{316\text{-}323}$ IP-MS (WashU) and Simoa (mean difference: 0.22, limits of agreement: -0.18 to 0.61), and (**C-D**) $NfL_{316\text{-}323}$ IP-MS (WashU) and $NfL_{316\text{-}323}$ IP-MS/MS (UCL) (mean difference: 0.15, limits of agreement: -0.23 to 0.54). The solid grey line on all Bland-Altman plots represents the line of equality, with the bias between methods represented as the mean difference. Upper and lower limits of agreement are plotted as + 1.96 s and -1.96 s respectively. (**E**) A plot of Log NfL measurements for comparison of IP-MS/MS (UCL) and Simoa is provided in supplement to the Bland-Altman analysis presented in Figure 3. Abbreviations: IP-MS, immunoprecipitation - mass spectrometry; IP-MS/MS, immunoprecipitation - tandem mass spectrometry; NfL, neurofilament light chain; s, standard deviation; Simoa, single-molecule array; UCL, University College London; WashU, Washington University in St Louis.

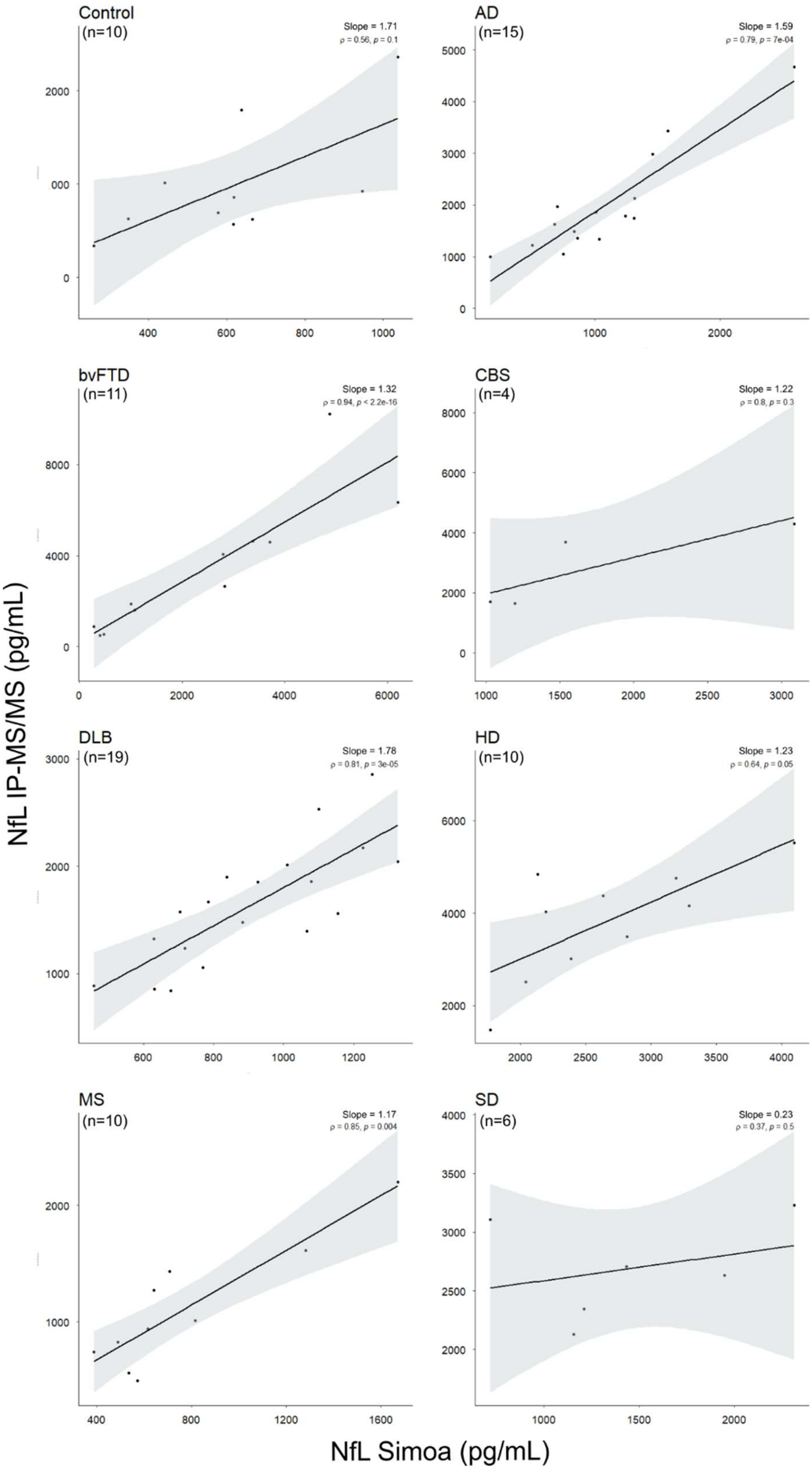

Supplementary Figure 4. Correlation of IP-MS/MS (UCL) and Simoa measures of NfL across clinical groups show very high correlation between assays for bvFTD, MS, DLB, CBS and AD.

Scatterplots of NfL concentration as measured by IP-MS/MS (UCL) vs Simoa are plotted for each clinical diagnostic group. Shading around the linear regression line represents 95% confidence intervals. Spearman's ranked

correlation coefficients and significance are noted on each individual plot and the slope of the linear regression is stated. Abbreviations: AD, Alzheimer's disease; bvFTD, behavioural variant frontotemporal dementia; CBS, corticobasal syndrome; DLB, dementia with Lewy bodies; HD, Huntington's disease; IP-MS, immunoprecipitation-mass spectrometry, IP-MS/MS, immunoprecipitation - tandem mass spectrometry; MS, multiple sclerosis; NfL, neurofilament light chain; SD, semantic dementia; Simoa, single-molecule array; UCL, University College London.

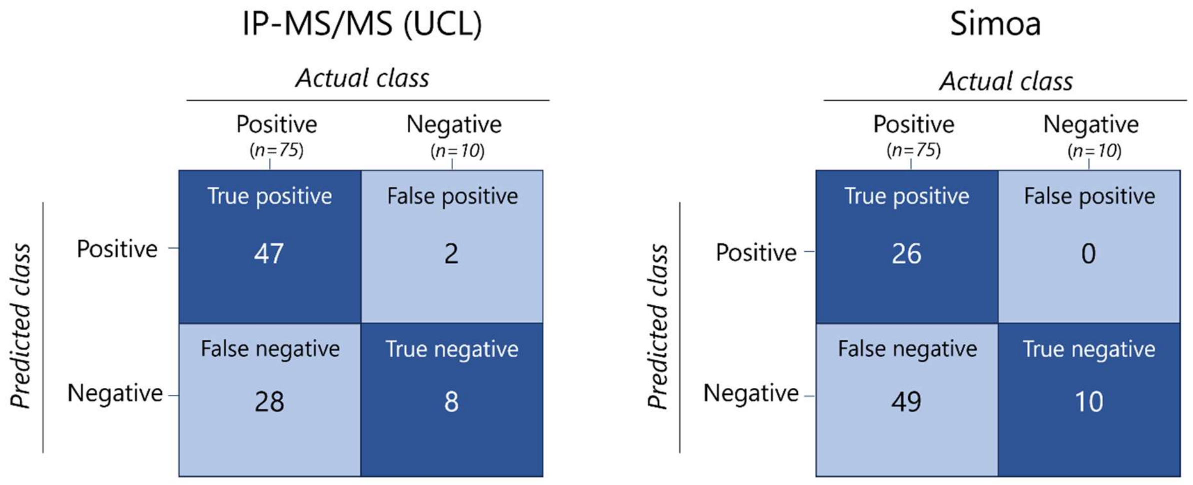

Assay	Sensitivity (%)	Specificity (%)	Positive predictive value (%)	Negative predictive value (%)	Accuracy (%)	Precision (%)
IP-MS/MS (UCL)	63	80	96	22	65	63
Simoa	35	100	100	17	42	35

Supplementary Figure 5. Confusion matrices for IP-MS/MS (UCL) and Simoa methods based on current age-related NfL clinical cut off values.

Confusion matrix analysis was performed to assess and compare method performance based on current clinical cut off values for CSF NfL concentrations. Actual class positive or negative assignment was based on clinical diagnosis of study participants at time of sampling as either those with a neurodegenerative disease or as a healthy control respectively. Predictive class positive or negative assignment was based on current age-related clinical cut off concentrations for normal CSF NfL. Abbreviations: IP-MS/MS, immunoprecipitation – tandem mass spectrometry; UCL, University College London; Simoa, single-molecule array.
